# Caveolin-1 and -2 in airway epithelium: expression and in situ association as detected by FRET-CLSM

**DOI:** 10.1186/1465-9921-7-108

**Published:** 2006-08-11

**Authors:** Gabriela Krasteva, Uwe Pfeil, Marek Drab, Wolfgang Kummer, Peter König

**Affiliations:** 1 Institut für Anatomie und Zellbiologie, University of Giessen Lung Center, Justus-Liebig-Universität Giessen, Germany; 2 Max-Planck Institute for Infection Biology, Department of Molecular Biology, Berlin, Germany

## Abstract

**Background:**

Caveolae are involved in diverse cellular functions such as signal transduction, cholesterol homeostasis, endo- and transcytosis, and also may serve as entry sites for microorganisms. Hence, their occurrence in epithelium of the airways might be expected but, nonetheless, has not yet been examined.

**Methods:**

Western blotting, real-time quantitative PCR analysis of abraded tracheal epithelium and laser-assisted microdissection combined with subsequent mRNA analysis were used to examine the expression of cav-1 and cav-2, two major caveolar coat proteins, in rat tracheal epithelium. Fluorescence immunohistochemistry was performed to locate caveolae and cav-1 and -2 in the airway epithelium of rats, mice and humans. Electron-microscopic analysis was used for the identification of caveolae. CLSM-FRET analysis determined the interaction of cav-1α and cav-2 in situ.

**Results:**

Western blotting and laser-assisted microdissection identified protein and transcripts, respectively, of cav-1 and cav-2 in airway epithelium. Real-time quantitative RT-PCR analysis of abraded tracheal epithelium revealed a higher expression of cav-2 than of cav-1. Immunoreactivities for cav-1 and for cav-2 were co-localized in the cell membrane of the basal cells and basolaterally in the ciliated epithelial cells of large airways of rat and human. However, no labeling for cav-1 or cav-2 was observed in the epithelial cells of small bronchi. Using conventional double-labeling indirect immunofluorescence combined with CLSM-FRET analysis, we detected an association of cav-1α and -2 in epithelial cells. The presence of caveolae was confirmed by electron microscopy. In contrast to human and rat, cav-1-immunoreactivity and caveolae were confined to basal cells in mice. Epithelial caveolae were absent in cav-1-deficient mice, implicating a requirement of this caveolar protein in epithelial caveolae formation.

**Conclusion:**

These results show that caveolae and caveolins are integral membrane components in basal and ciliated epithelial cells, indicating a crucial role in these cell types. In addition to their physiological role, they may be involved in airway infection.

## Background

Caveolae are omega-shaped invaginations of the plasma membrane measuring 50 to 100 nm in diameter. They are found in numerous cell types such as type I pneumocytes, endothelial cells, adipocytes, fibroblasts, smooth muscle cells, cardiac and striated muscle cells [1]. Caveolar formation is dependent on the expression of caveolins. Three caveolins (cav) are known. Cav-1 and cav-2 are widely expressed, whereas cav-3 is thought to be restricted to muscle cells [2]. Cav-1 is expressed in two isoforms, cav-1α and cav-1β, exhibiting a cell type-specific distribution (endothelial vs. alveolar type-1 cells) in the alveolar region [3].

Caveolae are involved in diverse cellular functions such as organizing signal transduction mechanisms, endocytosis and intracellular transport [2]. Several pathogenic microorganisms selectively use caveolae to enter cells [4]. After accumulation in the caveolae, they are delivered to the endoplasmatic reticulum bypassing the classical endosome-lysosome trafficking and thereby preventing inactivation [5,6]. It has been shown that the infectivity of C-type human adenovirus can be greatly reduced by the expression of a dominant negative cav-1 mutant in plasmocytic cells [7], indicating that caveolae are involved in this process. In addition, it was recently shown for Chlamydia pneumoniae that it co-localizes intracellularly with cav-1 and cav-2 after infection, and a role of these proteins for the developmental cycle of Chlamydiae is discussed [8]. Also, the human coronavirus 229E that is known to induce respiratory tract infections enters cells via a caveolae dependent mechanism [9,10].

Although the airway epithelium serves as entry site for microbes, fulfils functions that are associated with caveolae such as endo- and transcytosis, and harbors receptors that are associated with caveolae [1], the expression of caveolins, their interaction, and the presence of caveolae in tracheal and bronchial epithelial cells have not yet been determined. Interestingly, the presence of "vesicles that sometimes are connected with the membrane" has earlier been described at the electron-microscopic level in mouse basal cells [11]. Moreover cav-1 and cav-2 were detected in cell lines derived from bronchial epithelium [12], pointing to the presence of caveolae in the airway epithelium.

Both cav-1 and cav-2 exhibit a similar expression, but seem to have different functions. Cav-1 is sufficient to drive caveolar formation [13]. In general, it is thought that cav-2 alone is not sufficient for caveolae formation, and the absence of caveolae in cav-1-deficient mice is associated with marked reduction in cav-2 levels [14]. In contrast, although caveolae are still present in cav-2 deficient mice, these mice show the marked pathological alveolar phenotype of cav-1 deficient mice [15]. This indicates that cav-2, although not able to form caveolae on its own, has profound influences on caveolar function. Since a selective association of cav-2 but not cav-1 was described with Chlamydia species other than Chlamydia pneumoniae it is likely that both proteins can have divergent functions during infectious processes making it necessary to examine the presence and localization of both proteins.

In view of these facts, it is pivotal to gain insight in the cellular expression of caveolae and caveolins in bronchial and tracheal epithelium. We therefore examined the expression of cav-1 and cav-2 on the mRNA and protein level, determined the distribution of cav-1α, cav-1β, and cav-2 by immunohistochemistry and examined the presence of caveolae by electron microscopy. To address the molecular composition of caveolae, we determined the molecular association of cav-1α and cav-2 in tracheal epithelial cells in tissue sections by double-labeling indirect immunofluorescence combined with confocal laser scanning microscopy (CLSM) and fluorescence resonance energy transfer (FRET) analysis.

## Methods

### Animals

This study was performed on 1) Wistar rats (150–250 g) of either sex, kept either under standard laboratory conditions or under specified pathogen-free (SPF) conditions, and 2) cav-1-deficient mice [14] and the corresponding C57/Bl6 wild-type mice that were kept under SPF conditions. The animals were held according to the German guidelines for the care and use of laboratory animals. They were killed by inhalation of an overdose of isoflurane (Abbott, Wiesbaden, Germany).

### RT-PCR

Total RNA from abraded tracheal epithelial cells of adult Wistar rats (n = 6) was isolated by using the RNeasy method according to the manufacturer'sprotocol (Qiagen, Hilden, Germany). The epithelial cells from the trachea were abraded using cotton swabs that were carefully rolled over the epithelial layer. Contaminating DNA was degraded using 1 U DNase-I (Invitrogen, Karlsruhe, Germany) per μg of total RNA, and reverse transcription was done for 50 min at 42°C using 200 U Superscript II reverse transcriptase (Invitrogen) per μg of RNA. RT-PCR was performed by adding 1 μl cDNA, 0.5 μl of each gene-specific intron-spanning primer pair for cav-1 or cav-2 (20 pM; MWG Biotech, Ebersberg, Germany, Table 1), 2.5 μl 10 × PCR buffer II (100 mM Tris-HCl, 500 mM KCl, pH 8.3), 2 μl MgCl_2 _(15 mM), 0.5 μl dNTP (10 mM each), 0.1 μl AmpliTaqGold polymerase (5 U/μl; all reagents from Applied Biosystems, Darmstadt, Germany) and 17.9 μl H_2_O. Cycling conditions were 12 min at 95°C,40 cycles with 30 s at 95°C, 30 s at 59°C,30 s at 72°C, and a final extension at 72°C for 7 min. Control reactions included the absence of DNA template and the absence of reverse transcriptase. Primers for β-2-microglobulin (β-MG) were used as a positive control for efficiency of RNA isolation and cDNA synthesis. The PCR products were separated by electrophoresis on a 2% TRIS-acetate-EDTA agarose gel.

**Table 1:** Oligonucleotide primers for cav-1, cav-2 and β-MG (β-2-microglobulin) in RT-PCR analysis

Gene	GenBank accession NO	Primer	Product length (bp)	Position of amplified DNA (bp)
cav-1	Z46614.1	Forward: CAGCATGTCTGGGGGTAAAT Reverse: TGCTTCTCATTCACCTCGTCT	123	25–147
cav-1	Z46614.1	Forward: GGCTAGCTTCACCACCTTCA Reverse: GTGCAGGAAAGAGAGGATGG	121	165–285
cav-2	BC062059.1	Forward: TGTTTCTAGCCATCCCCTTG Reverse: ACCATGAGGCAGGTCTTCAC	106	392–497
cav-2	BC062059.1	Forward: CCTACAGCCACCACAGTGTC Reverse: GGTTCTGCGATCAGATCCTC	127	176–302
β-MG	NM_012512	Forward: TGTCTCAGTTCCACCCACCT Reverse: GGGCTCCTTCAGAGTGACG	191	147–337

### Real time RT-PCR

Total RNA was isolated from abraded tracheal epithelial cells of rats (n = 6, SPF; n = 3, standard conditions) and reverse-transcribed as described above. Real-time PCR was performed in an I-Cycler (Bio-Rad, Munich, Germany) using a QuantiTec SYBR Green PCR kit (Qiagen). Primer sets for cav-1 and -2 amplifying the sequences corresponding to nucleotides 25–147 and 392–497, respectively, were used (Table 1). The PCR conditions included initial denaturation in one cycle of 10 min at 95°C followed by 40 cycles of 20 s at 95°C, 20 s at 59°C, and 20 s at 72°C. All analyses were done in triplicate. As a basis for the relative mRNA quantification, the mean cycle thresholds (CT) for cav-1 and cav-2 were calculated. The corresponding threshold cycles of the target gene were subtracted from mean β-MG-CT according to:

ΔCT = CT_target gene _– CT_β-MG_

The relative expression (RE) of cav-2 compared to that of cav-1 was calculated as follows:

RE_cav-2 _= 2^ΔΔCTcav-2^, where

ΔΔCT_cav-2 _= ΔCT_cav-1 _- ΔCT_cav-2._

The PCR products were analyzed by electrophoresis on a 2% TRIS-acetate-EDTA agarose gel.

### Laser-assisted microdissection and subsequent RT-PCR

Laser-assisted microdissection (using a MicroBeam System, P.A.L.M. Microlaser Technologies, Bernried, Germany) was used to isolate epithelial cells from cryosections of tracheae of rats (SPF, n = 6; normal conditions, n = 2). Serial cryosections (6 μm) were collected on membrane slides (P.A.L.M. Microlaser Technologies), previously radiated with UV-light (254 nm) for 30 min. For each cup, the amount equal to 50% of the epithelium of a transverse section of a trachea was collected within 2 h after preparing the sections. RNA isolation and purification were performed using RNeasy Micro Kit (Qiagen) according to the manufacturer's protocol, but omitting the DNA digestion step. Ten μl RNA were incubated at 70°C for 10 min. RT-mix was added (2 μl 10 × PCR buffer II, 100 mM Tris-HCl, 500 mM KCl, pH 8.3; 4 μl MgCl_2_, 25 mM; 1 μl dNTPs, 10 mM; 1 μl Random Hexamers, 50 mM; 0.5 μl RNAse Inhibitor, 20 U/μl; 1 μl MuLV Reverse Transcriptase, 50 U/μl; 0.5 μl H_2_O; all reagents from Applied Biosystems). RNA was reverse-transcribed for 75 min at 43°C, followed by inactivation of the reverse transcriptase by heating the RNA samples for 5 min at 99°C. For subsequent PCR, 4 μl cDNA, 2.5 μl 10 × PCR buffer II, 2 μl MgCl_2 _(15 mM), 0.5 μl dNTPs (10 mM), 0.5 μl of each primer (20 pM; primer sets spanning the region 25–147 for cav-1 and 392–497 for cav-2), 0.2 μl AmpliTag Gold polymerase (5 U/μl, all reagents from Applied Biosystems) and 14.8 μl H_2_O were applied. Cycling conditions were 4 min at 95°C, 50 cycles with 20 s at 95°C, 20 s at 59°C, 20 s at 73°C, and a final extension at 73°C for 7 min. To control for smearing of RNA during the cutting procedure, areas of dried O.C.T. compound (Sakura, Zoeterwoude, The Netherlands) similar in number and size to the samples of picked epithelial cells and directly adjacent to the luminal side of the tracheal epithelium were applied. Control reactions for each primer pair included the absence of template. The PCR products were separated by electrophoresis on a 2% TRIS-acetate-EDTA agarose gel. Sequencing of the PCR products was done by MWG Biotech.

### Antibodies

Antibodies and their sources were as follows: anti-caveolin-1α (anti-cav-1α; immunohistochemistry (IHC) 1:400, Western blotting (WB) 1:500), polyclonal from rabbit (sc-894; Santa Cruz Biotechnology, Heidelberg, Germany); anti-caveolin-1αβ (anti-cav-1αβ; IHC 1:200; WB 1:500), monoclonal from mouse (clone 2297; Transduction Laboratories, Heidelberg, Germany); anti-caveolin-2 (anti-cav-2; IHC 1:200), monoclonal from mouse (clone 65; Transduction Laboratories); anti-endothelial nitric oxide synthase (anti-eNOS; IHC 1:100), monoclonal from mouse (clone 3; Transduction Laboratories); anti-surfactant protein D (anti-SP-D; IHC 1:100), monoclonal from mouse (clone VI F11, Dianova, Hamburg, Germany), anti-villin, polyclonal from rabbit (IHC 1:2,000, [16]). Secondary antibodies used in this study for immunohistochemistry were: FITC-conjugated donkey anti-mouse-lg, F(ab')_2 _fragments (1:200; Dianova), Cy3-conjugated donkey anti-rabbit-Ig (1:2,000; Chemicon, Temecula, CA, USA), Cy5-conjugated donkey anti-rabbit-Ig, F(ab')_2 _fragments (1:50; Dianova), and Cy3-conjugated donkey anti-mouse-Ig (1:1,000; Dianova). Secondary antibodies used in this study for Western blotting were: horseradish peroxidase-conjugated goat anti-rabbit-IgG or horseradish peroxidase-conjugated goat anti-mouse-IgG (both 1:10,000; Pierce, Rockford, USA).

### Electron microscopy

For conventional electron microscopy, tracheae of Wistar rats (normal conditions, n = 2), cav-1 deficient mice (SPF, n = 2), and C57/Bl6 mice (SPF, n = 3) were prepared as follows: The vascular system was flushed via the ascending aorta with a rinsing solution containing heparin (2 ml/l; 10,000 U; Ratiopharm, Ulm, Germany), polyvinylpyrrolidone (25 g/l, MW 40.000; Roth, Karlsruhe, Germany) and procaine hydrochloride (5 g/l; Merck, Darmstadt, Germany) pH 7.4, followed by the fixative consisting of 1.5% glutardialdehyde and 2% paraformaldehyde (PFA) in 0.1 M phosphate buffer (pH 7.4). Tracheae and bronchi were dissected, stored for additional 5 h in the same fixative, washed in 0.1 M TRIS-HCl buffer, osmicated for 1 h in aqueous 1% OsO_4_, washed (3 × 15 min) in 0.05 M maleate buffer (pH 5.2), stained en block for 1 h in 1% uranyl acetate in 0.05 M maleate buffer (pH 6.0), washed again (3 × 5 min) in 0.05 M maleate buffer (pH 5.2), dehydrated in ascending concentrations of ethanol and embedded in Epon. Sections of approximately 80 nm thickness were cut on an ultramicrotome (Reichert Ultracut E, Leica, Bensheim, Germany) stained with alkaline lead citrate and examined in an EM 902 transmission electron microscope (Zeiss, Jena, Germany).

### Immunohistochemistry

Unfixed tissue of Wistar rats, cav-1-deficient mice (SPF, n = 2), wild-type mice (SPF, n = 2), and 10% neutral formalin-fixed and paraffin-embedded human bronchi (n = 4) were used for immunohistochemical analysis. Lungs from rats and mice were inflated via the trachea with O.C.T. compound diluted with an equal amount of 0.1 M phosphate buffer (pH 7.4), orientated on a piece of filter paper, and shock-frozen in melting isopentane. Cryosections (10 μm) were cut, fixed (either with acetone at -20°C and air dried for 10 min or with 4% PFA for 20 min and washed) and incubated for1 h in 5% normal goat serum containing 5% BSA in 0.005 M phosphate-buffered saline (PBS). Primary antibodies were diluted in 0.005 M phosphate buffer containing 0.01 % NaN_3 _and 4.48 g/l NaCl and applied overnight at room temperature. These antibodies were appliedeither singly or in combination for double-labeling immunofluorescence. Primary antibody combinations were as follows: mouse anti-cav-1αβ/rabbit anti-cav-1α; mouse anti-cav-2/rabbit anti-cav-1α; mouse anti-eNOS/rabbit anti-cav-1α; mouse anti-SP-D/rabbit anti-cav-1α; mouse anti-cav-2/rabbit anti-villin. After a washing step, Cy3-conjugated donkey anti-rabbit-Ig was applied for 1 h and after a second washing step the slides were incubated with FITC-conjugated F(ab')_2 _donkey anti-mouse-Ig. Sections wererinsed, postfixed for 10 min in 4% PFA, rinsed again and coverslipped with carbonate-buffered glycerol (pH 8.6). Sections from human bronchi (6 μm) were deparaffinated and incubated with anti-cav-1α and anti-cav-2 antibody as described above. Slides were evaluated with an epifluorescence microscope (Zeiss, Jena, Germany) using appropriate filter sets and with a confocal laser scanning microscope (Leica-TCS SP2 AOBS;Leica, Mannheim, Germany).

Specificity of the anti-cav-1α antibody was validated by incubation of cryosections from cav-1 deficient mice. The specificity of the anti-cav-1αβ and anti-cav-2 antibodies was previously shown by other groups in experiments with cav-2 deficient mice [17]. Here, we characterized these antibodies by Western blotting. Additional controls included omission of the primary antibodies.

### Western blot

For Western blot analysis, abraded tracheal epithelial cells of Wistar rats (n = 5), hearts and lungs from Wistar rats, lungs from cav-1-deficient mice and from wild-type mice (each n = 2) were homogenized by a mixer mill (MM 300, Qiagen) with lysis buffer containing 10 mM Tris (pH 7.5), 50 mM NaCl, 1% Triton X-100, 60 mM octylglucoside (Sigma-Aldrich Chemie GmbH, Munich, Germany), and one Complete Mini Protease Inhibitor Cocktail tablet (Roche Diagnostics GmbH, Mannheim, Germany) per 10 ml buffer. After incubation of the protein solutions at 4°C for 1 h, they were centrifugedfor 5 min at 14,000 rpm. Equal amounts of proteins for each tissue were applied as judged by staining of gels with Simply BlueTM Safe Stain (Invitrogen, Carlsbad, USA) stained gels. Ten μl appropriately diluted protein solution and 2 μl of 5 × sample buffer (320 mM Tris-HCl, pH 6.8, 5% SDS, 50% glycerol, 0.25 mg/ml bromphenol blue and 1% β-2-mercaptoethanol) were boiled for 5 min at 95°C. The samples were subjected to 15% SDS-PAGE under reducing conditions and subsequently transferred to a nitrocellulose membrane (Bio-Rad) by semidry blotting. The nitrocellulose membranes were stained with Ponceau S to confirm the protein transfer from the gels to the membrane. After subsequent washing in 25 mM Tris-buffered saline with 0.05% Tween-20 (TTBS), the unspecific binding sites were saturated by incubation with 10% non-fat dry milk in TTBS for1 h at room temperature. The membrane was incubated overnight at 4°C with anti-cav-1α, -1αβ and -2, primary antibodies, diluted in 5% non-fat dry milk in TTBS. The secondary antibodies were diluted in 2.5% non-fat dry milk in TTBS and incubated for 1 h at room temperature. Super Signal West Pico Chemiluminescence Substrate (Pierce) was used for visualization. Controls were done by omitting the primary antibody, and the specificity of the anti-cav-1α and anti-cav-2 antibodies was verified in lung homogenates from cav-1-deficient mice.

### Fluorescence Resonance Energy Transfer (FRET)

FRET is a nonradiative energy transfer between two fluorophores (a donor and an acceptor) that can be detected only if the two fluorophores are less than 10 nm apart. We used FRET combined with CLSM and double-labeling indirect immunofluorescence as a technique for measuring close spatial association of proteins in tissue sections [18]. Cav-1 and cav-2 were labeled using conventional indirect double-labeling immunofluorescence technique (anti-cav-1α from rabbit labeled with Cy5-conjugated secondary reagent, anti-cav-2 antibody labeled with Cy3-conjugated secondary reagent). Both primary antibodies were applied simultaneously. After a washing step, Cy3-conjugated donkey anti-mouse-Ig was applied for 1 h and after a second washing step the slides were incubated with Cy5-conjugated F(ab')_2 _donkey anti-rabbit-Ig. For control of the species-specificity of the secondary reagents, only the anti-cav-1 antibody and both secondary antibodies were applied. FRET was quantified by the acceptor photobleaching method using a CLSM (TCS-SP2 AOBS, Leica). In this method, FRET is detected by measuring the intensity of fluorescence of the donor before and after bleaching of the acceptor. The CLSM settings were as follows: Detection of Cy3: 52% laser power at 543 nm, detection at 555–620 nm; Cy5: 20% laser power at 633 nm, detection at 639–738 nm. A region of interest was photobleached 10 times at 100% activity using the 633 nm laser and maximal zoom to destroy the acceptor fluorophore (Cy5) and the change in Cy3 signal (ΔIF) was determined in the photobleached area where

ΔIF = D_DA _– D_DB_,

D_DA _is the fluorescence intensity of the donor after photobleaching of the acceptor, and D_DB _is the fluorescence intensity of the donor before photobleaching of the acceptor.

### Statistical analyses

Differences among experimental group and control group in the FRET-experiments were analysed with the Kruskal-Wallis test followed by Mann-Whitney test using SPSS software, version 11.5.1 (SPSS GmbH Software, Munich, Germany), with p ≤ 0.05 being considered as significant and p ≤ 0.01 as highly significant.

## Results

### RT-PCR

**Figure 1:**
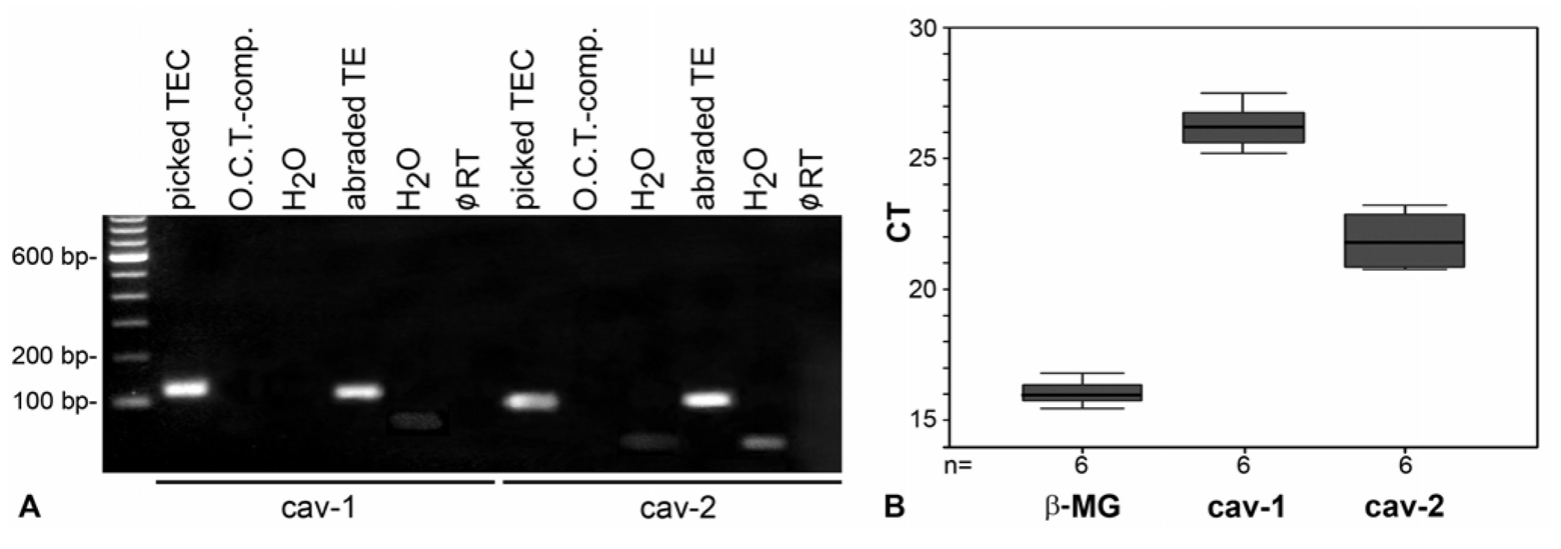
RT-PCR, rat. (A) Cav-1 and cav-2 mRNAs were detected in abraded tracheal epithelium (TE) and in microdissected (picked) tracheal epithelial cells (TEC) with primer sets spanning 25–147 bp and 392–497 bp within the coding region, respectively. Control reactions for each primer pair included the absence of template (H_2_0), absence of reverse transcriptase (Ø RT), and analysis of samples of dried O.C.T. compound that were located adjacent to the luminal side of the collected tracheal epithelial cells. (B) Real-time RT-PCR. CT values for β-MG, cav-1 and cav-2 derived from cDNA from abraded tracheal epithelial cells; n = number of animals.

RT-PCR analysis of total mRNA isolated from rat lungs and abraded tracheal epithelial cells revealed expression of cav-1 and cav-2. PCR products were of the expected size and the sequence was verified by sequencing. No bands were observed in control reactions that included absence of DNA template or the reverse transcriptase (Figure 1A).

We quantified the relative expression of cav-1 and cav-2 in tracheal epithelial cells in rats held under standard conditions or SPF conditions. In both groups, cav-2 expression was higher than that of cav-1. Cav-2 expression levels were 22.2 times higher than that of cav-1 under standard conditions and 25.8 times higher in animals housed under SPF conditions (Figure 1B).

### Laser-assisted microdissection

In accordance to the results obtained in abraded epithelial cells, mRNA for cav-1 was detected in 17/18 samples of microdissected tracheal epithelial cells collected from 5 rats (SPF). Cav-2 mRNA was present in all microdissected samples (n = 15, 4 SPF rats). The identity of the PCR products was validated by sequencing. No mRNA for cav-1 and cav-2 was detected in areas next to positive tissue that contained O.C.T. compound only, but no cells. This result indicates that the mRNA signal detected in the epithelium for cav-1 and cav-2 was not caused by contamination originating from caveolin-containing adjacent cells (Figure 1A).

### Western blot

**Figure 2:**
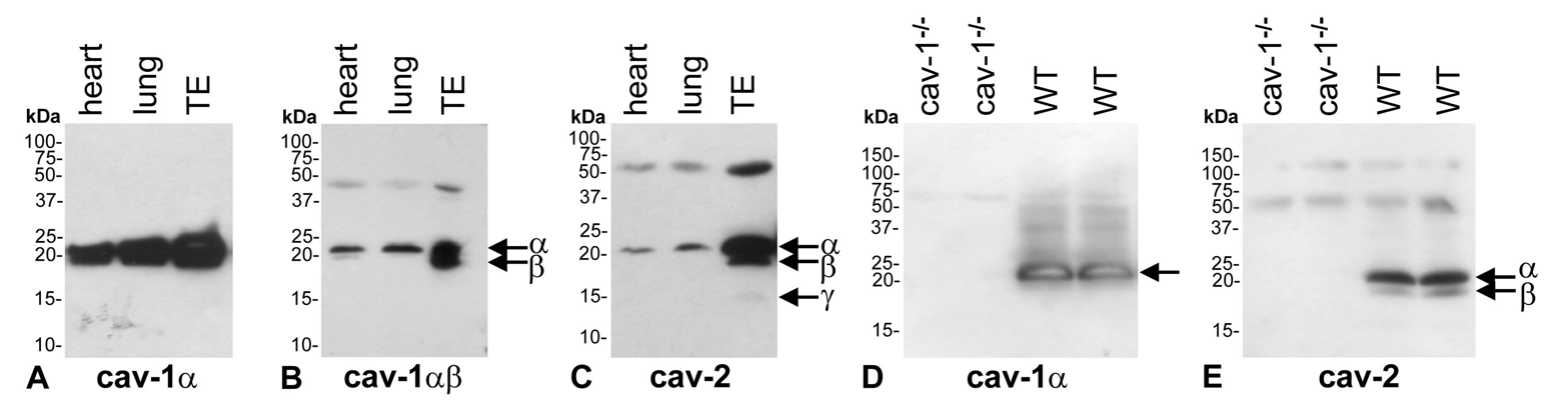
Western blotting. (A) A single 22 kD protein was detected in abraded rat tracheal epithelium (TE) with the affinity-purified anti-cav-1α antibody. (B) 22 kD (α) and 18 kD (β) cav-1 protein isoforms were detected in abraded rat TE with monoclonal affinity-purified anti-cav-1αβ antibody. (C) 21 kD (α) and 18 kD (β) bands and, close to the detection limit, a 15 kD (γ) band, corresponding to the three known cav-2-isoforms, were detected with monoclonal anti-cav-2 antibody in abraded TE. (A-C) Heart and lung homogenates served as controls. (D) In contrast to wild-type mice (WT), no cav-1α was detected in lung homogenates of cav-1-deficient mice (cav-1^-/-^). (E) Also, no cav-2 protein was detected in lung homogenates of cav-1-deficient mice. The 50 kD bands in all lanes were due to the secondary antibodies that detected the heavy chain of mouse IgG.

Using the anti-cav-1α antibody, we detected a single band of approximately 22 kD in rat abraded tracheal epithelial cells (Figure 2A). A band of 22 kD and a band of 18 kD, corresponding to cav-1β, were detected in abraded tracheal epithelial cells using an anti-cav-1αβ antibody (Figure 2B). Bands of the same molecular weight were detected in lung and heart homogenates. Using an antibody to cav-2, we detected a band of 15 kD (γ-isoform) that was close to the detection limit, an 18 kD band (α-isoform), and a 21 kD band (β-isoform) (Figure 2C). In lung homogenates of wild-type mice, cav-1α-immunoreactive bands of 22 kD, 37 kD, and 48 kD were detected, which were absent in lung homogenates from cav-1 deficient mice (Figure 2D). Using an antibody to cav-2 for Western blot analysis of lung homogenates from wild-type mice we detected 18 kD and 21 kD bands. No cav-2 bands were detected in lung homogenates from cav-1-deficient mice (Figure 2E).

### Immunohistochemistry

**Figure 3:**
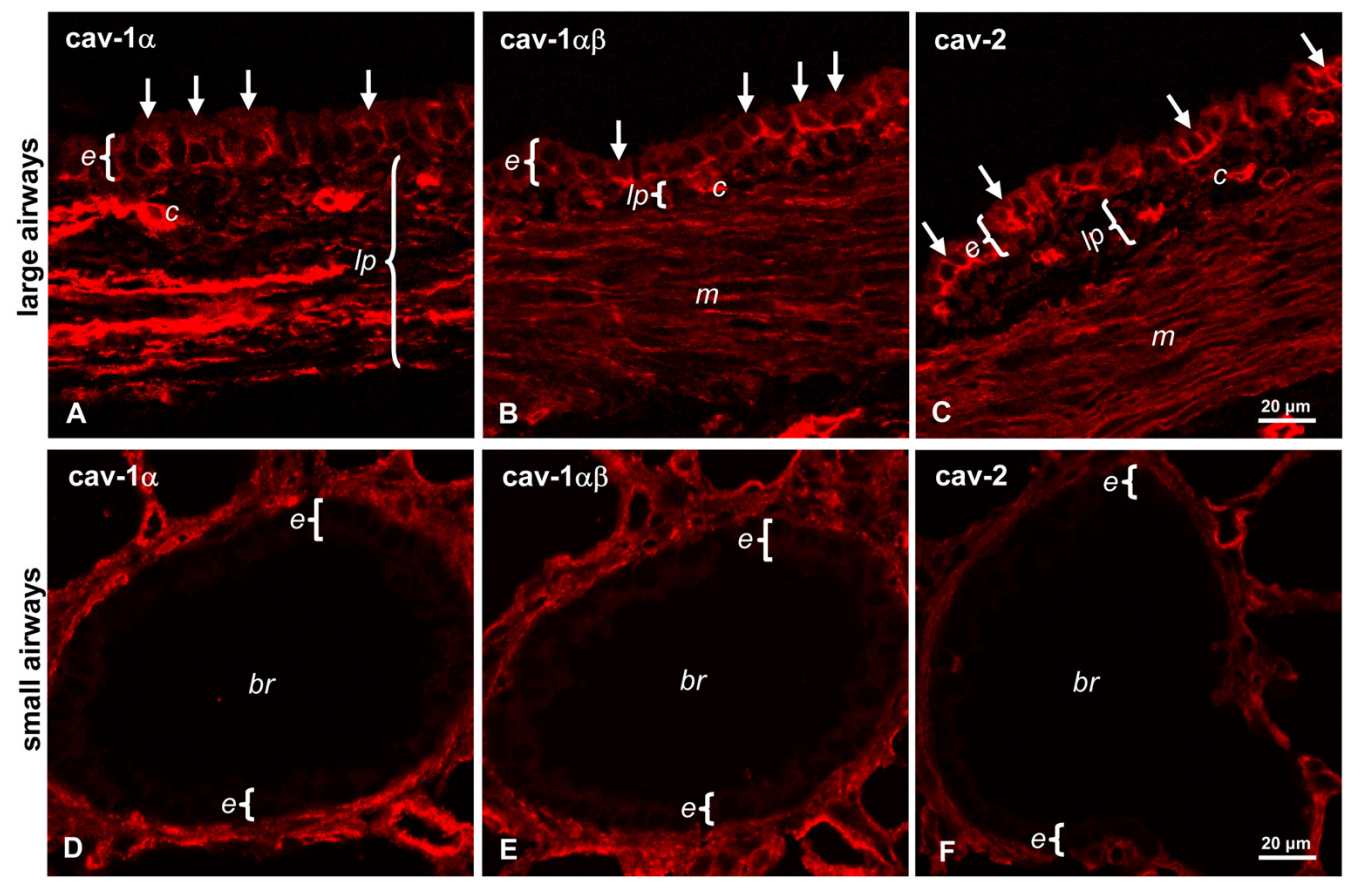
Immunohistochemistry, rat. (A-C) Large airways. (A) Epithelial cells (arrows) and cells of the lamina propria were immunoreactive for cav-1α. The labeling of the endothelial cells of capillaries (*c*) in the lamina propria was stronger compared to that of the epithelium. (B) Anti-cav-1αβ antibody labeled the same cell types as the anti-cav-1α antibody (arrows), however, with stronger intensity of the labeling in the epithelium compared to that in the lamina propria and the smooth muscle cells. (C) Epithelial cells (arrows) as well as cells of the lamina propria and smooth muscle cells were immunoreactive for cav-2. (D-F) Small airways, bronchioli (*br*). Endothelial cells and type I epithelial cells of the surrounding alveoli were immunoreactive for cav-1α, cav-1αβ and cav-2. No immunoreactivity was detected in the bronchiolar epithelium. Epithelium = *e*, smooth muscle cells = *m*, lamina propria = *lp*.

In rat, cav-1-immunoreactivity was detected in epithelial cells of the large airways (Figure 3A). In contrast to large cartilaginous airways, no cav-1-immunolabeling was detected in the epithelium of the small non-cartilaginous airways of the rat (Figure 3D). In addition, cav-1-immunoreactivity was observed in tracheal and bronchial smooth muscle cells, in vascular endothelial cells and in unidentified cells in the lamina propria, probably fibroblasts. In the alveolar region, we observed cav-1-immunoreactivity in endothelial cells and in type I alveolar epithelial cells.

**Figure 4:**
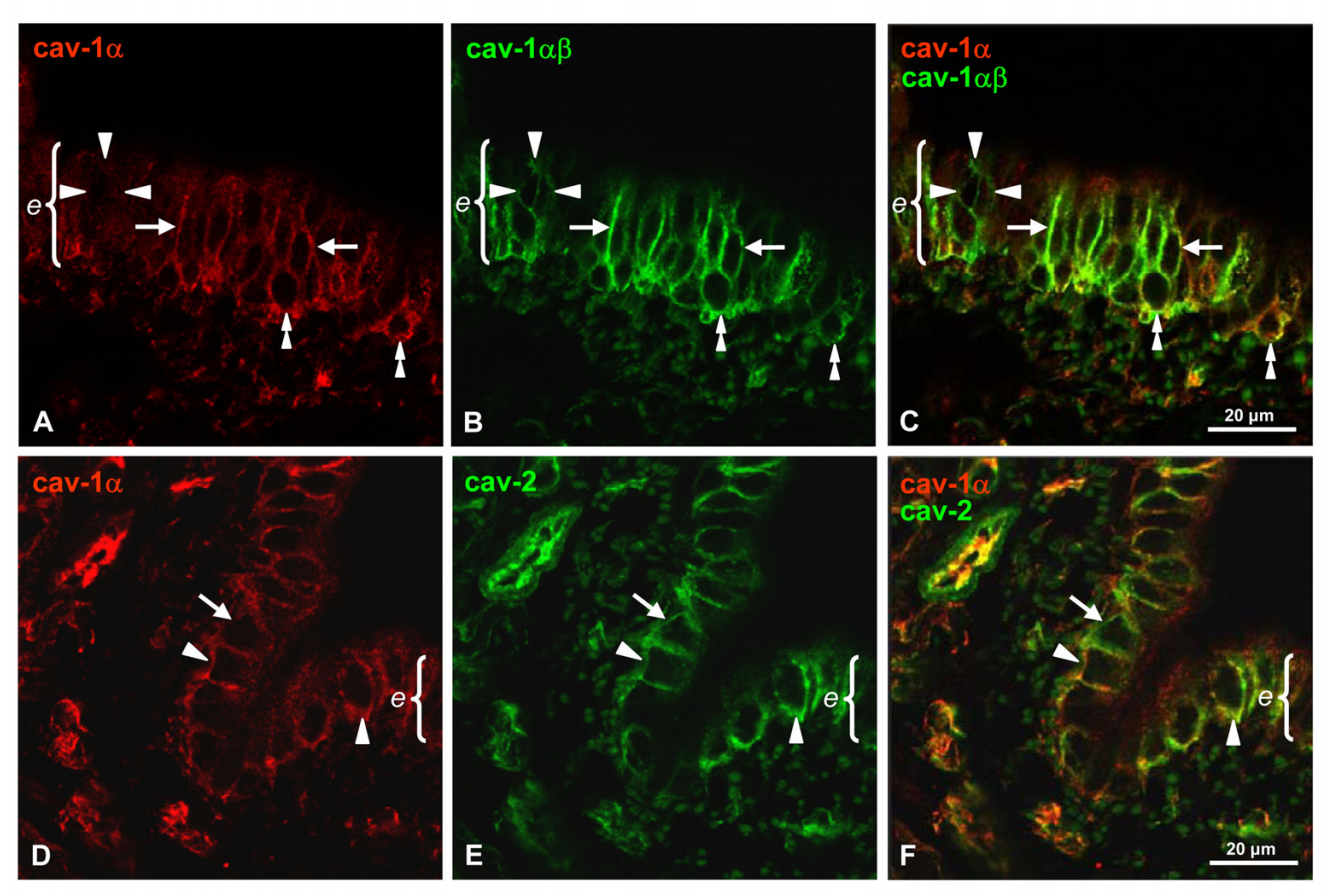
Double-labeling immunofluorescence, CLSM, large airways, rat. (A-C) Cav-1α- (A) and cav-1αβ-immunoreactivity (B) were co-localized in the basal cells (doubled arrowheads) and basolaterally in a subset of columnar epithelial cells (arrows). Some cells exhibited cav-1αβ- but not cav-1α-immunoreactivity (arrowheads). (D-F) Cav-1α (D) and cav-2 (E) were localized at the basolateral membrane. Co-localisation of cav-1α and cav-2 (F, arrowheads). We observed cells that were only cav-2-immunoreactive (F, arrows). Epithelium = *e*

Differences in the labeling patterns obtained with the anti-cav-1α and the anti-cav-1αβ antibody were noted. The labeling with the anti-cav-1α antibody was stronger in the endothelium, whereas labeling of the anti-cav-1αβ antibody was stronger in the epithelium (cf. Figures 3A, B). Also, the number of the epithelial cells that were immunoreactive for cav-1αβ was higher than that of the cells immunoreactive for cav-1α (Figures 4A–C).

Cav-2-immunoreactivity was found in the same cell types as cav-1-immunoreactivity. Interestingly, not all of the cells that showed cav-2-immunofluorescence were cav-1α-immunoreactive (Figures 4D–F). The cav-2-immunolabeling of the epithelium was stronger than that of smooth muscle cells (Figure 3C). As for cav-1, no labeling for cav-2 was observed in the epithelial cells of the small bronchi (Figure 3F).

**Figure 5:**
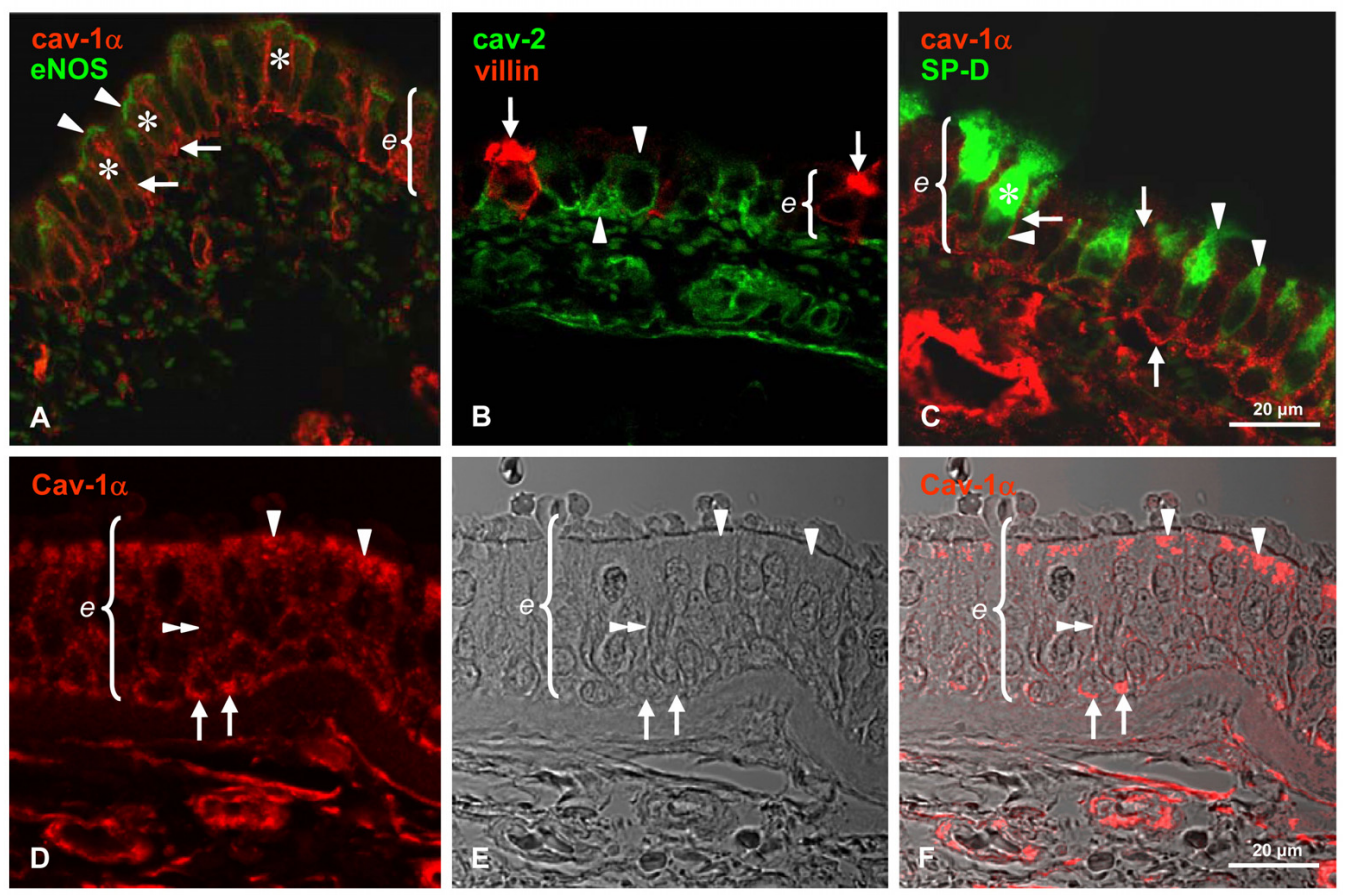
Cav-immunoreactive epithelial cell types. (A-C) Rat. (A) Cav-1α and eNOS double-labeling. Cav-1α-immunoreactivity (red, arrow) was found in ciliated cells (asterisks), labeled by anti-eNOS antibody (green, arrowhead). (B) Brush cells, labeled with anti-villin antibody (red, arrows) were not immunoreactive for cav-2 (green, arrowhead). (C) Cav-1α-immunoreactivity (red, arrow) in columnar epithelial cells and basal cells. Secretory cells were labeled with anti-SP-D antibody (green, arrowheads). In few secretory cells (asterisk), we could not determine definitely if they express cav-1α, since the cav-1α-immunoreactivity was localized at the cell membrane and SP-D-immunoreactivity was localized in the cytoplasm. (D-F) Human bronchus. Ciliated (arrowheads) and basal cells (arrows) were immunoreactive for cav-1α. Cell types are identified by their typical morphology as seen in the CLSM transmitted light mode. Punctate labeling for cav-1α was localized at the basolateral membrane (doubled arrowhead) and in the apical area of the ciliated cell beneath the luminal plasma membrane (arrowheads). Epithelium = *e*

Throughout the rat trachea and large bronchi, we found cav-1α- and cav-1αβ-immunoreactivity 1) in the ciliated cells, identified by their typical morphology and by immunolabeling with a monoclonal antibodyto eNOS [19] (Figure 5A), and 2) in the basal cells, identified by their typical morphology. Unlike ciliated cells, brush cells immunoreactive for villin were not labeled for cav-2 (Figure 5B). Most of the secretory cells that were labeled with anti-SP-D [20] were not cav-1-immunoreactive (Figure 5C).

In human bronchi, the ciliated and the basal cells were immunoreactive for cav-1α (Figures 5D–F). The anti-cav-2 antibody gave no signal in paraffin-embedded human tissue. Punctate cav-1α labeling was localized at the basolateral membrane and in the apical area of the ciliated cells underneath the luminal plasma membrane.

**Figure 6:**
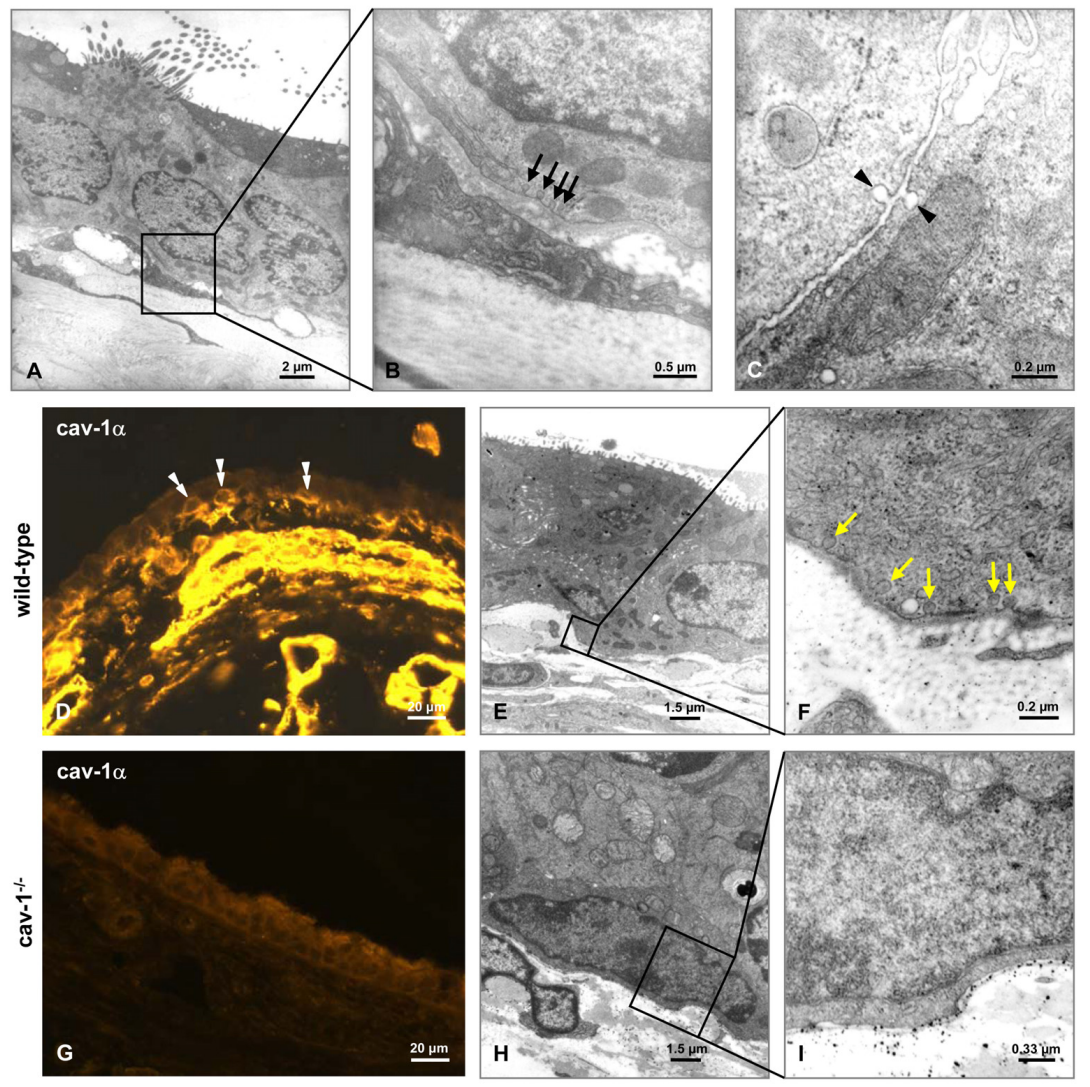
(A-C) Electron microscopy, rat. Caveolae were identified in the basal (A and B, arrows) and in the lateral membrane (C, arrowheads) of ciliated cells in the rat trachea. (D-I) Electron-microscopy and immunohistochemistry, wild-type mouse. Caveolae were present only in the basal cells of the mouse trachea (E and F, arrows) and only this cell type was immunoreactive for cav-1α (D, arrowheads). (G-I) In tracheal epithelial cells of cav-1 deficient mice, neither cav-1α-immunoreactivity (G) nor caveolae (H and I) were found in the basal cells.

In the mouse respiratory epithelium, cav-1α-immunoreactivity was confined to basal cells (Figure 6D). The specificity of the cav-1-immunostaining was confirmed by the absence of cav-1α-immunolabeling in cav-1-deficient mice (Figure 6G).

### Electron microscopy

In agreement with the immunohistochemical results, we observed caveolae at the basolateral cell membrane of the ciliated cells and at the cell membrane of the basal cells in rat trachea (Figures 6A–C). Caveolae were more frequent in basal cells. In agreement with the immunohistochemical findings in the mouse trachea, caveolae were only found in the basal cells (Figures 6D–F). Basal cells were devoid of caveolae in tracheae of cav-1-deficient mice (Figures 6G–I).

### CLSM-FRET in tissue sections

**Figure 7:**
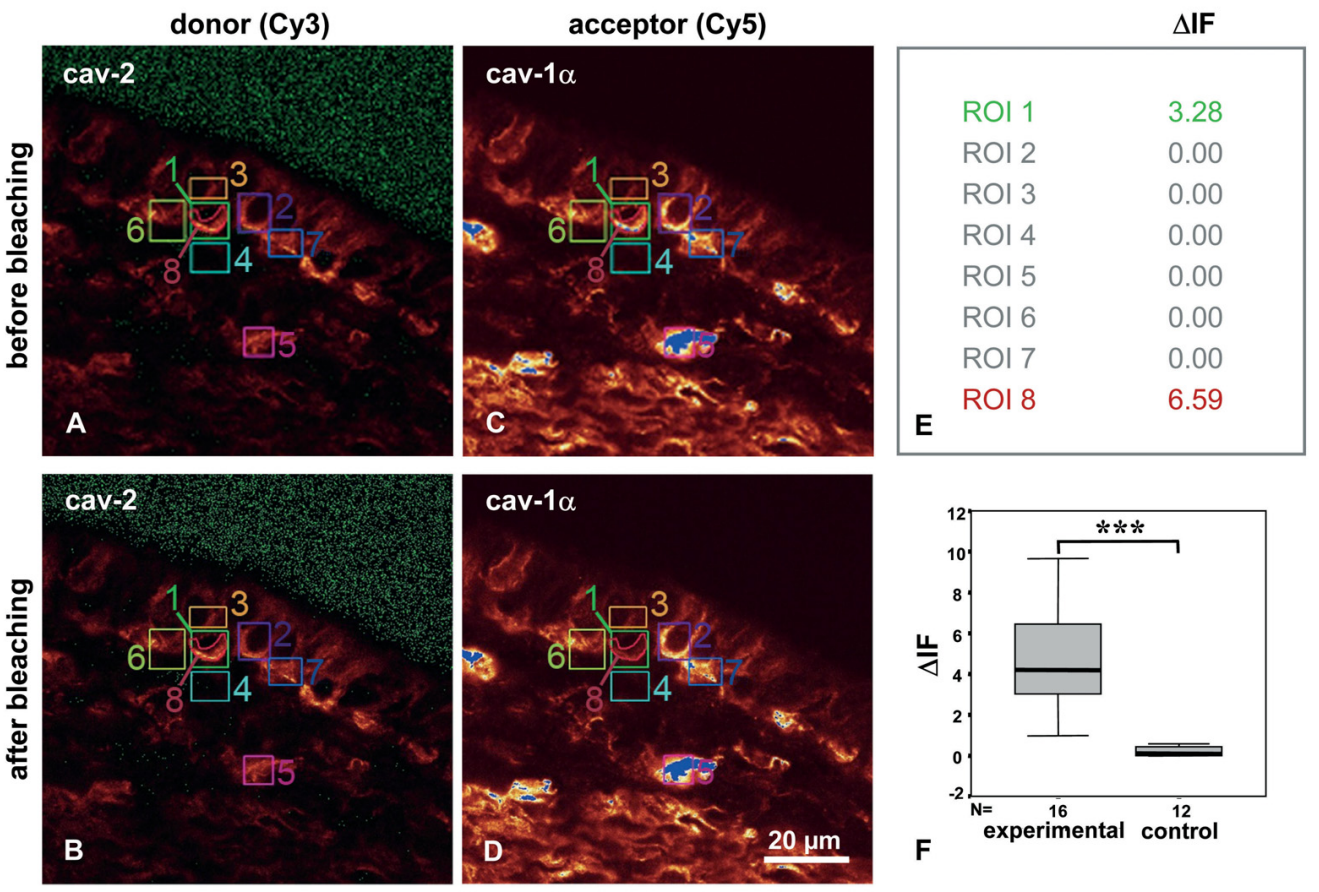
Detection of close association of cav-1 and cav-2 in epithelial cells by double-labeling indirect immunofluorescence and FRET in tissue sections of the rat trachea and large bronchi. Images of donor (cav-2 labeled with Cy3-conjugated secondary antibody, A-B) and acceptor (cav-1α labeled with Cy5-conjugated secondary antibody, C-D) fluorescence. Cy5 was bleached in a region of interest 1 (compare ROI 1 in C and D). E: ΔIF for each ROI. ROI 1: bleached area (compare C and D). ROI 8: freehand encircled region of the basolateral plasma membrane of the epithelial cells in bleached area. ROI 2–7: control areas outside the bleached area. F: ΔIF in the area of the basolateral membrane in experimental group (4 animals) compared to control group. *** p ≤ 0.001, Mann-Whitney test. n = number of measurements. Boxplots: percentiles 0, 25, median, 75, 100.

Conventional indirect double-labeling immunofluorescence with subsequent FRET-CLSM analysis was conducted to determine whether cav-1 and cav-2 are in close apposition in airway epithelial cells in situ, thereby indicating an association of both proteins and formation of hetero-oligomers. Distinct increase of fluorescence (ΔIF) was observed in the bleached area with a median value of 2.72 (n = 16 regions of interest of tracheae obtained from 4 rats; Figure 7). A false-positive FRET signal that can be caused by cross-reactivity of secondary antibodies was excluded by applying both secondary antibodies to sections incubated with anti-cav-1 α antibody only (median ΔIF = 0.287). Since the caveolins are membrane proteins, we measured ΔIF in the region of the basolateral plasma membrane. ΔIF measured in this region was higher (ΔIF = 4.795) than that in the whole bleached area. The ΔIF measured in the controls in the same region was low (ΔIF = 0.55). The difference between ΔIF observed in the experimental group as compared to the corresponding controls was highly significant (p < 0.001, Mann-Whitney test; Figure 7F). No increase in the donor fluorescence (ΔIF) could be detected in the airway epithelial cells that were immunoreactive for cav-2, but not for cav-1α (data not shown).

## Discussion

The present study demonstrates for the first time the expression of cav-1 and -2 in epithelial cells of the trachea and large bronchi. Electron microscopy demonstrated that, indeed, ciliated and basal cells possess caveolae. It has been shown that muscarinic receptors as well as β-adrenergic receptors may translocate to caveolae upon agonist binding [21,22]. These receptors are involved in the regulation of ciliary function [23,24]. In addition, several proteins involved in Ca^2+^-dependent signaling processes, including the Ca^2+^-pump and Ca^2+^-ATPase, are localized to caveolae as shown in renal and intestinal epithelial cells [25]. Therefore, caveolae are likely to be involved in the fine-tuned regulation of epithelial cytosolic Ca^2+^-concentration and in regulation of ciliary function.

Several pathogenic microorganisms selectively use caveolae to enter cells [4]. Since the caveolae are localized at the basolateral surface of the ciliated cells, it is conceivable that they may be involved in the process of endocytosis of infectious agents after epithelial damage. Indeed, adenoviruses require damage of the integrity of the epithelia or of the tight junctions to get access to the basolateral membrane of the ciliated cells to be infectious [26]. Basal cells are much more susceptible to infection with adenoviruses [7,27]. Accordingly, we observed more caveolae in basal cells than in ciliated epithelial cells. It has been shown for the adenovirus2 that it can enter the cell via its receptor CAR that is also localized basolaterally in ciliated cells and in basal cells. Since adenovirus2-CAR is endocytosed via clathrin-coated pits, caveolae, at first sight, seem not to be important in this process. Nevertheless, another receptor for adenovirus2 is major histocompatibility complex (MHC) class 1. This also is localized in the basolateral membrane of ciliated cells and in basal cells. MHC class 1 is also the receptor for simian virus 40 that is endocytosed via caveolae. This indicates a role for caveolae in the infection of airway epithelium by these adenoviruses. Furthermore, the infectivity of C-type human adenovirus can be greatly reduced by the expression of a dominant negative cav-1 mutant in plasmocytic cells indicating that caveolae can serve as an alternative entry site for these viruses [28]. In addition, it was recently shown for Chlamydia pneumoniae that it co-localizes with cav-1 and cav-2 in the cytosol in HeLa cells after cellular entrance [8], indicating a role for caveolins in infectious processes after microbial entry.

Since the loss of cav-1 was accompanied with loss of caveolae in tracheal epithelial cells in cav-1-deficient mice, cav-1 is required for the formation of caveolae in these cells. In addition, the stabilization and the transport of cav-2 to the plasma membrane are dependent on the expression of cav-1 [29,30]. Our results from FRET experiments also prove that cav-1α and cav-2 are closely associated in ciliated and in basal cells, indicating that both proteins are involved in the formation of caveolae. Interestingly, we found considerably higher expression of cav-2 mRNA than cav-1 mRNA, indicating that cav-2 could have roles independent from cav-1 once it has reached the plasma membrane. Indeed, it has been shown for cav-2 that it associates with chlamydial inclusions independently of cav-1 [8].

We observed differences in the labeling intensities for cav-1α and cav-1αβ among cell types. Cav-1α-immunoreactivity was stronger in endothelial cells compared to epithelial cells. In contrast, cav-1αβ-labeling was stronger in the epithelium than in the endothelium of the large airways. In line with this observation, Western blots showed a strong protein expression of cav-1β in abraded tracheal epithelium but not in lung and heart homogenates. Cav-1β is less efficient in the formation of caveolae [31], which could be an explanation for the lower number of caveolae found in epithelial cells compared to endothelium where the α-isoform is predominantly expressed [3]. The β-isoform of cav-1 is derived from an alternative translational starting site that creates a protein truncated by 32 amino acids [32] and lacks the phosphorylation site tyr 14. This site has been shown to be phosphorylated upon stimulation of cultured cells by epithelial growth factor (EGF), leading to neoformation of caveolae [33]. Since EGF is considered as a key factor for repair of the bronchial epithelium [34], caveolae containing the α-isoform might be involved in this process. It is tempting to speculate that certain caveolae may exist that contain predominantly the β-isoform. Such caveolae might be involved in other signaling cascades. If both isoforms are present in caveolae, cav-1β could be a negative regulator for signaling cascades relying on the phosphorylation of cav-1α.

We found caveolin-1 and -2 only in the large airways of mice and rats, limiting caveolar function to larger airways in these species. It has to be kept in mind, however, that many human intrapulmonary bronchi are considerably larger than the rat trachea. Since we have found cav-1 also in human intrapulmonary bronchi, caveolins are likely to be present throughout a substantial part of the human bronchial tree.

## Conclusion

In summary, we conclude that ciliated and basal cells of the trachea and large bronchi possess caveolae resulting from an association between cav-1α and cav-2. Since caveolae are implied in a variety of different functions in other cell types, they are likely to be important also for these functions in the airway epithelium.

## Abbreviations

BSA, bovine serum albumin

cav, caveolin

CLSM, confocal laser scanning microscopy

CT, cycle threshold

ΔIF, change in Cy3 signal

EGF, epidermal growth factor

eNOS, endothelial nitric oxide synthase

FRET, fluorescence resonance energy transfer

IHC, immunohistochemistry

MHC, major histocompatibility complex

β-MG, β-2-microglobulin

PFA, paraformaldehyde

ROI, region of interest

RE, relative expression

SP-D, surfactant protein D

SPF, specified pathogen-free

TE, tracheal epithelium

TEC, tracheal epithelial cells

TTBS, Tris-buffered saline with Tween-20

WB, Western blotting

## Competing interests

The author(s) declare that they have no competing interests.

## Authors' contributions

PK and GK conceived and designed the study. GK performed the immunohistochemical analysis, the Western blot experiments and the laser-assisted microdissection with subsequent RT-PCR analysis. GK and PK performed the FRET analysis. UP and GK carried out the quantitative RT-PCR. WK, GK, and PK analyzed the electron microscopic specimens. MD generated and genotyped the cav-1 deficient mice. PK and GK performed the statistical analysis. The manuscript was drafted by GK, WK, and PK.
